# Comparative analysis of chronic rhinitis patient profiles during autumn pollen season between grassland and non-grassland cities in North China

**DOI:** 10.1186/s13223-021-00591-w

**Published:** 2021-10-11

**Authors:** Xu Xu, Long Qin, Lei Ren, Chengshuo Wang, Yuan Zhang, Luo Zhang

**Affiliations:** 1 Department of Otolaryngology Head and Neck Surgery, Beijing Tong Ren Hospital, Capital Medical University, Beijing, China; 2 Beijing Key Laboratory of Nasal Diseases, Beijing Institute of Otolaryngology, No. 17, Hou Gou Hu Tong, Dong Cheng District, 100005 Beijing, People’s Republic of China; 3 Department of Allergy, Beijing Tong Ren Hospital, Capital Medical University, Beijing, China; 4 The Second Affiliated Hospital of Baotou Medical College, Baotou Medical College, Baotou, China

**Keywords:** Chronic rhinitis, Allergic rhinitis, Autumn pollen season, Baotou, Beijing

## Abstract

**Background:**

The symptoms of patients with respiratory disease are influenced by local environmental factors. The incidence of allergic rhinitis in grassland areas was significantly higher than that in non-grassland areas. We aimed to compare the profiles of chronic rhinitis patients obtained during the autumn pollen season in Baotou (grassland city) and Beijing (non-grassland city), China.

**Methods:**

Questionnaire surveys and allergen testing were conducted on 1170 and 1232 patients with chronic rhinitis visiting the Second Affiliated Hospital of Baotou Medical College and Beijing Tongren Hospital, respectively, during the autumn pollen period. Information regarding medical history, severity of symptoms, and diagnosis and treatment was collected.

**Results:**

More patients with moderate to severe chronic rhinitis and asthma (both, *P* < 0.001) were present in Baotou than in Beijing. Mugwort was the most abundant allergen in both regions, but the number of patients sensitized to outdoor allergens in Baotou was higher than that in Beijing (*P* < 0.001). Indoor allergens in Beijing represented a considerable proportion of allergens, especially dust mites (33.4%). For patients with allergic rhinitis, nasal congestion, nasal itching, and runny nose were more severe in Baotou than in Beijing (*P* < 0.001). In both Baotou and Beijing, allergy (*P* < 0.001 vs. *P* = 0.004) and combined asthma (*P* = 0.049 vs. *P* = 0.005) were common factors affecting the severity of the clinical symptoms chronic rhinitis. In Baotou, age (*r*_*s*_ = 0.195, *P* < 0.001) and family allergy history (*P* = 0.010) were also associated with symptom severity. Although significantly more patients in Baotou received oral antihistamines, nasal corticosteroids, and surgical treatment than in Beijing (*P* < 0.001), the number of people receiving allergy immunotherapy in Baotou was lower (*P* = 0.004) and post-treatment symptom control was worse (*P* < 0.001) that that in Beijing.

**Conclusions:**

During the pollen period, there were significant differences in the allergen spectrum between Baotou and Beijing. Allergy and combined asthma were common factors affecting the severity of clinical symptoms. Patients in Baotou presented with more severe clinical symptoms that were not satisfactorily managed due to the impact of pollen exposure, inconsistent access to care, and differing treatment modalities.

**Supplementary Information:**

The online version contains supplementary material available at 10.1186/s13223-021-00591-w.

## Introduction

Chronic rhinitis (CR) is a common inflammatory disease of the nasal mucosa. Its clinical manifestations include nasal congestion, nasal itching, runny nose, and sneezing. It is estimated that CR affects about 30% of the global population and causes a significant social and economic burden [1]. Clinically, CR is categorized as allergic rhinitis (AR) and non-AR (NAR) based on the etiology. Patients with AR and NAR often show similar nasal symptoms, and the difference is based on the result of allergen sensitization.

AR is a global health problem that affects 10–40% of the population worldwide, causing major illness and disability [2, 3]. A longitudinal study from 18 major cities in China showed that the self-reported prevalence of AR in adults increased by 6.5% in 6 years [4]. Mugwort and house dust mites are the two most commonly inhaled allergens in Asia [5]. In North China, more than 50% of individuals with respiratory allergy are allergic to Artemisia pollen [6]. Zhang et al. [7] showed that there is a strong correlation between the pollen concentration in the ambient air and the number of AR diagnoses and treatment. Epidemiological studies have shown that the prevalence of AR presents significant regional differences due to regional environmental and climatic conditions [8]. The incidence of AR in grassland areas was significantly higher than that in non-grassland areas. In China, the average self-reported prevalence of AR in adults is 17.6%; in Beijing, this prevalence is 20.2% [4]. In contrast, the AR prevalence in grassland areas can be as high as 32.4% based on epidemiological data and 18.5% based on allergen screening [8]. As a representative of the Eurasian grassland, the Inner Mongolia grassland is characterized by an abundance of pollen and diverse species; the high local prevalence of AR is attributed to climatic and environmental factors, particularly the large amount of pollen exposure [8–10]. Thus far, no studies have examined the influence of pollen exposure on the clinical characteristics of local patients with CR, particularly the symptom severity and quality of life associated with varying intensities of pollen exposure.

Baotou and Beijing are both cities in North China. Baotou is a grassland area located in the central and western part of the Inner Mongolian Autonomous Region, while Beijing is a non-grassland city. These cities represent evident environmental differences in climatic variables, airborne pollen allergen intensity and period of exposure. Moreover, as the capital of China, Beijing is more developed than Baotou (third-tier city) in terms of the economy and medical level. The purpose of this study was to compare the clinical profiles of patients with CR during the autumn pollen season in Baotou and Beijing. We intended to elucidate the influence of differences in pollen allergen intensity and exposure period on the disease severity. We also investigated the differences in treatment status for these patients to help set up an effective allergy management strategy in the studied regions.

## Methods

### Study region

This study was jointly conducted by the Second Affiliated Hospital of Baotou Medical College in Inner Mongolia Autonomous Region, China and the Beijing Tongren Hospital in Beijing, China. We continuously included outpatients who volunteered to participate in the survey during the autumn pollen period of 2019 in both regions. Patients were eligible to join the study if they meet the following criteria: (1) age 18–60 years; (2) permanent resident of the Baotou or Beijing areas (lived in the region for more than half a year); (3) experienced two or more nasal symptoms (nasal congestion, nasal itching, runny nose, sneezing) in the past year; and (4) had received oral antihistamines, nasal corticosteroids, surgery, allergy immunotherapy or other treatments (oral hormones, antibiotics, mucus promoting agents, nasal irrigation, etc.) for nasal symptoms. We collected the serum samples and conducted allergen testing when the participants visited the hospital for treatment during the autumn pollen period; the participants completed the questionnaire survey at the same time. The diagnosis of AR was based on the latest AR episode and its impact on asthma (Allergic Rhinitis and its Impact on Asthma, ARIA) [3]. AR was confirmed by the presence of symptoms induced by exposure to an allergen shown to produce a serum allergen specific-IgE (sIgE), and the remaining patients were diagnosed with NAR.

Under the guidance of specialist doctors and nurses, a questionnaire survey was conducted in both regions. The questionnaire has been provided in an additional file [see Additional file 1]. The information collected included basic demographics (age and sex), severity of nasal symptoms in the past year, incidence of combined asthma, family history of allergies, smoking history, impact of rhinitis on life in the past year, and all previous medical history.

The prevalence of AR and NAR in rural areas in northern China is reported to be 16.78% and 24.60%, respectively [11]; thus, we set the test level α at 0.05, test power 1-β at 0.80, and calculate the required sample size as 806 cases. Considering that the prevalence of AR in urban areas is higher than that in rural areas [12], along with the consumption of sample size, we increased the sample size by 20%, with the final estimated required sample size being 967 cases. Incomplete questionnaires were deemed invalid. The number of questionnaires finally included in the statistical analysis in Beijing and Baotou were 1232 and 1170, respectively.

### Selection of the autumn pollen season

For each area, the pollen population shows a constant pattern of seasonal changes. Based on data describing the total pollen concentration in the environment provided by the Baotou and Beijing Meteorological Bureaus, the start of pollen season was defined when the pollen count was 5 pollen grains/m^3^ per day for more than 3 consecutive days, while the end of pollen season was defined when the pollen count fell to < 10 pollen grains/m^3^ per day for more than 3 consecutive days [13]. Based on the daily pollen concentration from 2019, data collected from patients from 20 July to 10 October 2019 in Baotou and Beijing from 13 August to 2 October 2019 were selected for this study.

### Definitions used in this study

According to the AR classification recommended by ARIA, the AR was diagnosed as intermittent when the symptoms occurred < 4 days/week or disease course was < 4 weeks; persistent when symptoms occurred for > 4 days/week and disease course was > 4 weeks. A diagnosis of mild AR was defined as normal performance of (1) sleep; (2) daily activities, sports, and entertainment activities; (3) work and study. Symptoms were defined as moderate or severe when they satisfied one or more of the following: (1) inability to sleep normally; (2) daily activities, physical exercise, entertainment were affected; (3) inability able to work or study normally; (4) presence of bothersome symptoms. To meet the needs of further statistical analysis, this classification method was also applied to the NAR population. The diagnosis of asthma adhered to the corresponding international diagnostic standards [14].

The availability for diagnostic reagents for the skin prick test is limited in China, which hinders doctors from using it for preliminary screening for allergens. Thus, in the present study, serological allergen-specific IgE tests were performed for all the enrolled patients, and the Immuno-CAP-1000 in vitro allergen detection system by Thermo Fisher Scientific was used to determine serum allergen sIgE. A serum sIgE level of ≥ 0.35kUA/L was considered allergen-positive [15]. Outdoor allergens included trees, ragweed, mugwort, and humulus, and indoor allergens included dust mites, house dust, cat hair, dog hair, cockroaches, and mold.

The subjective assessment of symptoms of patients with CR was based on the scores of four nasal symptoms (nasal congestion, nasal itching, runny nose, and sneezing), and were classified as follows: 0 = none, 1 = mild, 2 = moderate, and 3 = severe. The total nasal symptom score (TNSS) was used to evaluate the total score of the four nasal symptoms (range, 0–12).

The improvement of the symptoms of the enrolled patients after treatment was evaluated using the patient Global Impression of Change, and was scored as follows: 0 = symptoms were aggravated, 1 = no control over symptoms, 2 = minor control over symptoms, 3 = substantial control over symptoms, and 4 = total control over symptoms [16]. We defined patients with scores of 0 and 1 as those with “symptoms uncontrolled after treatment,” and patients with scores of 2 to 4 as those with “symptoms controlled after treatment.”

### Statistical analysis

Statistical analyses were performed using the SPSS 26.0 software (IBM Corp.). The Kolmogorov–Smirnov test was used to evaluate the normality of the data. Descriptive statistics were used to study the population characteristics and general information. Non-normally distributed continuous variables were expressed as interquartile ranges, and the Mann–Whitney U and Chi-square tests were performed to analyze the differences in clinical characteristics between patients in Baotou and Beijing. The chi-square test was used to compare the allergen profiles of patients with AR in the two regions. The Mann–Whitney U test was used to analyze the difference in the severity of clinical symptoms between the AR population and the NAR population in the two regions. When evaluating the factors affecting the clinical symptoms of the patients in the two regions, a Spearman correlation analysis was performed on the continuous variable indicators of non-normal distribution, and the Mann–Whitney U test was used for the remaining binary variables. The chi-square test was used to compare the diagnosis and treatment of patients with CR in the two regions. A P-value of < 0.05 was considered statistically significant.

## Results

### Demographic and clinical characteristics

This study recruited patients with CR who were eligible for enrollment during the pollen period in two regions in 2019, including 1170 individuals in Baotou and 1232 in Beijing. The demographic and clinical characteristics of the patients enrolled in the two regions are shown in Table 1. There were no significant differences between the patients in the two regions in terms of age or sex. The number of patients with a disease course of < 1 year was significantly greater in Baotou than that in Beijing, and the number of patients with a disease course of 6–10 years was higher in Beijing than in Baotou (*P* < 0.001). The number of patients with intermittent and persistent CR was similar in Beijing, while intermittent CR was the dominant type in Baotou (accounting for 74.2%), with the number of these patients being significantly higher in Baotou than in Beijing (74.2% vs. 46.7%, *P* < 0.001). The number of patients with moderate to severe CR was significantly higher in Baotou than in Beijing (91.8% vs. 81.9%, *P* < 0.001). Allergen test results showed that the number of allergen-positive patients in Beijing was higher than that in Baotou (65.6% vs. 61.6%, *P* = 0.044), but the number of patients allergic to outdoor allergens in Baotou was higher than that in Beijing (89.9% vs. 75.5%, *P* < 0.001). Baotou had a significantly higher prevalence of concomitant asthma than that in Beijing (13.5% vs. 7.2%, *P* < 0.001). The total scores of the clinical symptoms of patients with CR in Baotou and Beijing were 8.0 (4.0–12.0) and 8.0 (6.0–9.0) respectively, and there was a statistically significant difference between the two regions (*P* < 0.001).

**Table 1:** Demographic and clinical characteristics of the study population

Characteristic	Baotou (N = 1170)	Beijing (N = 1232)	*P-*value
Age (years), median (IQR)	33.0 (25.0–47.0)	32.0 (28.0–37.0)	0.097
Sex (male/female), n (%)	584 (49.9%)/586 (50.1%)	614 (49.8%)/618 (50.2%)	0.970
Course of disease, n (%)			** < 0.001**
< **1 year**	328 (28.0%)	221 (18.0%)	
1–5 year	571 (48.8%)	636 (51.6%)	
** 6–10 year**	190 (16.2%)	269 (21.8%)	
> 10 year	81 (6.9%)	106 (8.6%)	
Persistent/intermittent, n (%)	302 (25.8%)/868 (74.2%)	657 (53.3%)/575 (46.7%)	** < 0.001**
Mild/moderate to severe, n (%)	96 (8.2%)/1074 (91.8%)	223 (18.1%)/1009 (81.9%)	** < 0.001**
Allergen, n (%)			
Positive/negative	721 (61.6%)/449 (38.4%)	808 (65.6%)/424 (34.4%)	**0.044**
Outdoor allergens/indoor allergens	648 (89.9%)/301 (41.7%)	610 (75.5%)/470 (58.2%)	** < 0.001**
With asthma, n (%)	158 (13.5%)	89 (7.2%)	** < 0.001**
TNSS, median (IQR)	8.0 (4.0–12.0)	8.0 (6.0–9.0)	** < 0.001**

Descriptive statistics were used to study population characteristics and general information. Non-normally distributed continuous variables were described using the interquartile range, and Mann–Whitney *U* test and Chi-square test were performed to analyze the differences in clinical characteristics between Baotou and Beijing. TNSS: total nasal symptom score. *P* < 0.05 is shown in bold

### Allergen spectrum

**Fig. 1:**
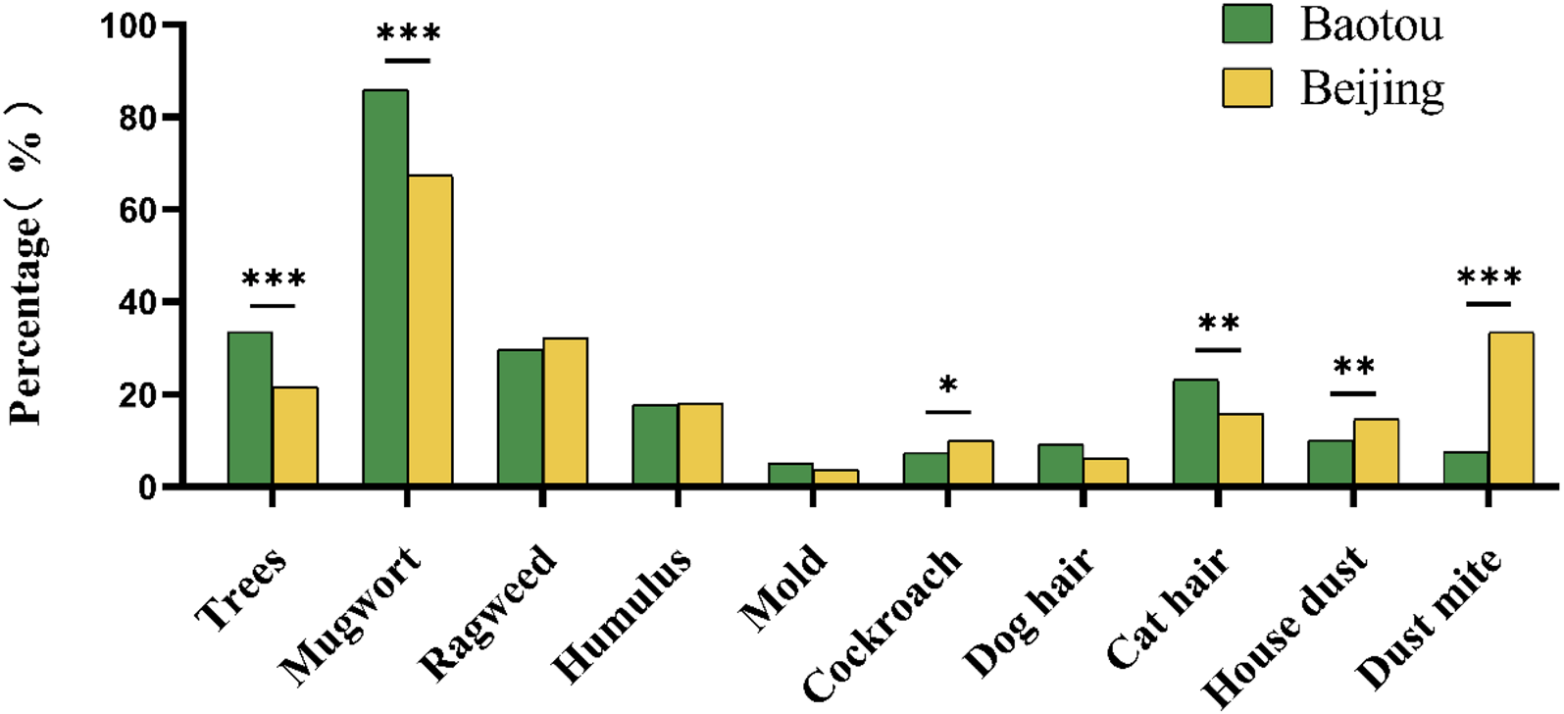
Comparison of allergen profiles of patients with AR in Baotou and Beijing. The chi-square test was used to compare the allergen profiles of patients with AR in the two regions. *AR* Allergic rhinitis. *P < 0.05; **P < 0.01, ***P < 0.001

**Fig. 2:**
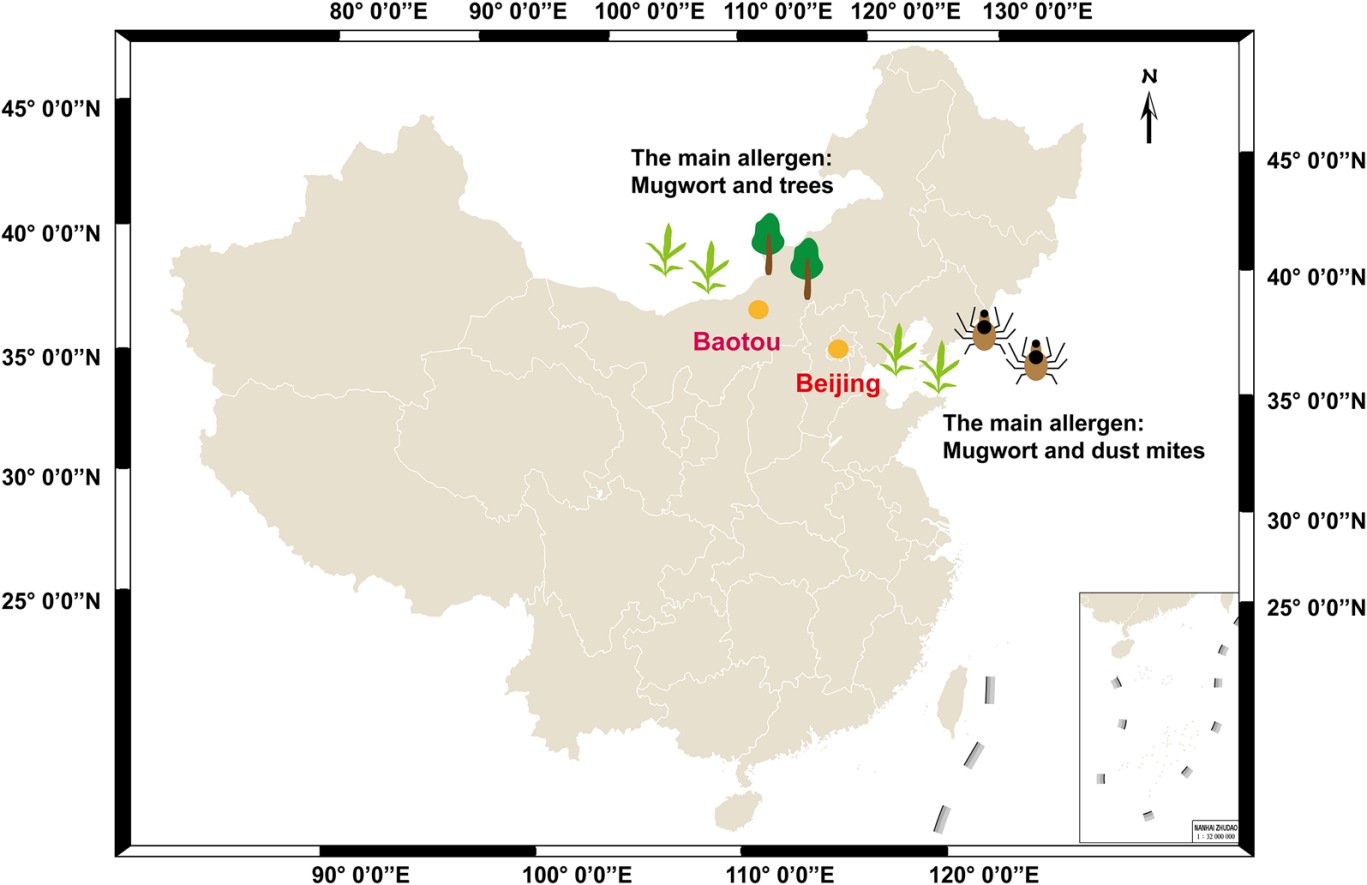
Distribution map of major allergens in Baotou and Beijing

Allergen testing was performed on all the participants in the two regions to define the allergen spectrum (Fig. 1) and major allergens (Fig. 2) of patients with AR in the two regions. Among the 10 most common allergens tested, mugwort was the predominant allergen in the pollen period for both regions. In Baotou, outdoor allergens (trees, mugwort, ragweed, and humulus) were the main allergens during the pollen period. In Beijing, dust mites also accounted for a considerable proportion allergens, and became the second largest allergen after mugwort. More patients were allergic to trees (*P* < 0.001), mugwort (*P* < 0.001), and cat hair (*P* < 0.01) in Baotou than in Beijing, while more patients were allergic to cockroaches (*P* < 0.05), house dust (*P* < 0.01), and dust mites (*P* < 0.001) in Beijing.

### Analysis of severity of clinical symptoms and risk factors

**Fig. 3:**
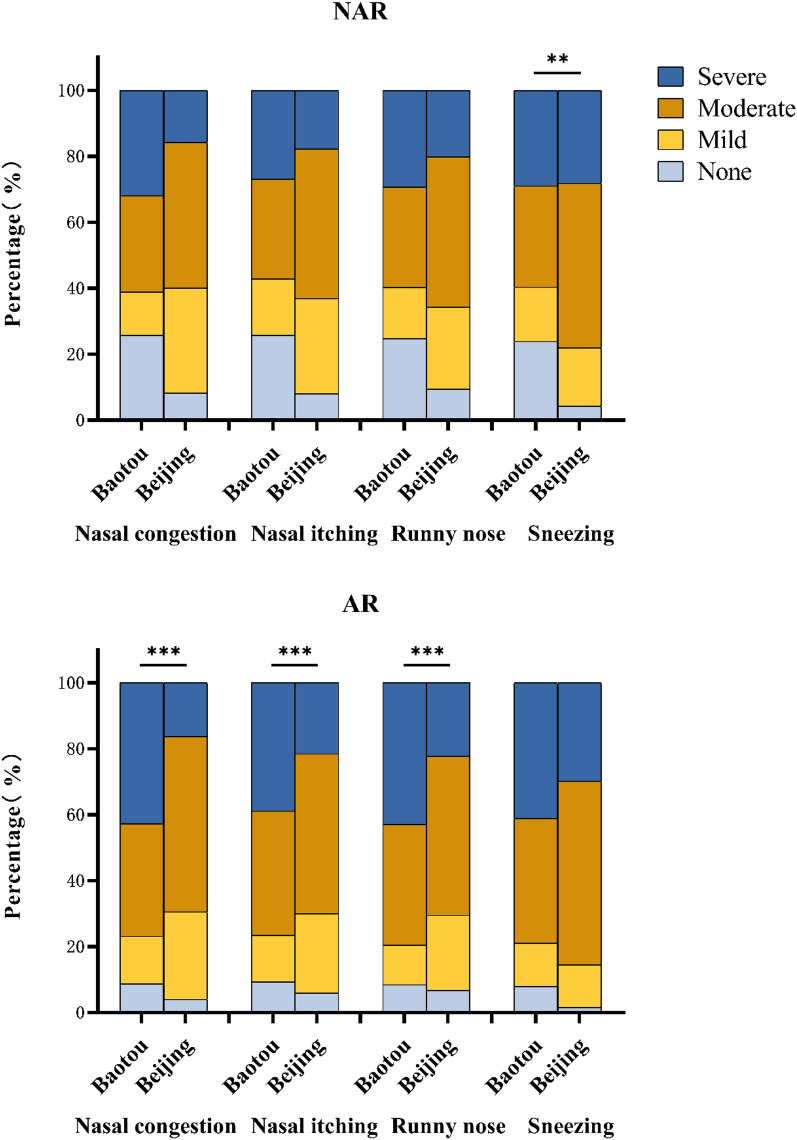
Comparison of severity of clinical symptoms between AR and NAR populations in Baotou and Beijing. The Mann–Whitney U test was used to analyze the difference in the severity of clinical symptoms between the AR and NAR population in the two regions. *AR* Allergic rhinitis, *NAR* nonallergic rhinitis. **P < 0.01, ***P < 0.001

We categorized the presence and severity of the four nasal symptoms (nasal blockage, nasal itching, runny nose, and sneezing) as none, mild, moderate, or severe, and subsequently compared the severity of the clinical symptoms of the AR and NAR populations in the two regions (Fig. 3). Among the AR population, the number of patients with the four symptoms scored as “severe” was higher in Baotou than that in Beijing. Among them, the symptoms of nasal congestion, nasal itching, and runny nose were more frequent (*P* < 0.001). Patients were more frequently categorized as having “mild to moderate” symptoms in Beijing compared to those in Baotou. This phenomenon was not observed among the NAR population.

We also analyzed the risk factors for the severity of clinical symptoms in both regions (Table 2). Allergy and combined asthma were common risk factors affecting the severity of clinical symptoms in the two regions (*P* < 0.05). In addition, for patients with CR in Baotou, age (*r*_*s*_ = 0.195, *P* < 0.001) and family history of allergies (*P* = 0.010) were also associated with the severity of clinical symptoms.

**Table 2:** Analysis of related factors on the severity of clinical symptoms of chronic rhinitis patients in Baotou and Beijing

Variable	Baotou		Beijing	
	*r*_*s*_	*P-*value	*r*_*s*_	*P-*value
Age	0.195	** < 0.001**	0.009	0.766
Sex	–	0.478	–	0.514
Allergic or not	–	** < 0.001**	–	**0.004**
Family history of allergy	–	**0.010**	–	0.990
Smoking history	–	0.062	–	0.074
With asthma	–	**0.049**	–	**0.005**

A Spearman correlation analysis was performed on the continuous variable indicators of non-normal distribution (age), and the Mann–Whitney *U* test was used for the remaining binary variables in order to evaluate the factors affecting the clinical symptoms of patients in the two regions. *P* < 0.05 is shown in bold

### Treatment status

We determined the treatment status of patients with CR in the two regions that received a diagnosis and treatment for their nasal symptoms (Table 3). In Baotou, the number of patients treated with oral antihistamines (32.6%vs. 25.3%, *P* < 0.001), nasal corticosteroids (33.9 vs. 18.4%, *P* < 0.001), and surgery (39.3 vs. 4.4%, *P* < 0.001) was significantly higher than that in Beijing, while in Beijing, more patients chose allergy immunotherapy (1.3 vs. 0.3%, *P* = 0.004) and other treatment (58.9 vs. 46.9%, *P* < 0.001) options. Although most patients with CR in Baotou were treated with oral antihistamines, nasal corticosteroids, and/or surgery, the symptom control after treatment was significantly worse than that in Beijing (50% vs. 77.4%, *P* < 0.001). In addition, 45.7% of patients with CR in Baotou had not visited a hospital in the past year, which was much a higher proportion than that in Beijing, where 86.3% of patients had 1–5 hospital visits per year (*P* < 0.001).

**Table 3:** Comparison of diagnosis and treatment of patients with chronic rhinitis in Baotou and Beijing

	Baotou (N = 1170)	Beijing (N = 1232)	*P* value
Treatment, n (%)			
Oral antihistamines	381 (32.6%)	312 (25.3%)	** < 0.001**
Nasal corticosteroids	397 (33.9%)	227 (18.4%)	** < 0.001**
Surgical treatments	460 (39.3%)	54 (4.4%)	** < 0.001**
Allergy immunotherapy	3 (0.3%)	16 (1.3%)	**0.004**
Other treatments	549 (46.9%)	726 (58.9%)	** < 0.001**
Symptoms controlled/uncontrolled after treatment	585 (50%)/585 (50%)	954 (77.4%)/278 (22.6%)	** < 0.001**
Number of visits in the past year (times/year), n (%)			** < 0.001**
0	535 (45.7%)	110 (8.9%)	
1–5	576 (49.2%)	1063 (86.3%)	
6–10	43 (3.7%)	47 (3.8%)	
> 10	16 (1.4%)	12 (1.0%)	

The chi-square test was used to compare the diagnoses and treatment of patients with chronic rhinitis in the two regions. *P* < 0.05 is shown in bold

## Discussion

CR is a common upper respiratory tract disease that can occur at the same time as other respiratory diseases and causes considerable economic and social burden [1]. As one of the most common diseases, AR has received considerable research interest globally, and NAR also affects about 200 million people worldwide [17, 18]. Pollen produced by gramineous plants is currently listed as the main air allergen. Studies conducted around the world have emphasized that differences in climate and environment will affect the concentration of pollen in the air, which has a major impact on allergic diseases [19]. This study collected clinical information on the autumn pollen exposure of CR patients in Baotou and Beijing, and compared and analyzed the differences in the clinical status of CR in the two regions, which provides significant reference for guiding the clinical treatment and intervention of patients with CR.

Our research shows that there were significant differences in the allergen spectrum between the Baotou and Beijing regions. This difference was closely related to differences in the environmental conditions between the two regions. Inner Mongolia has the largest grassland area in China. Compared with the Beijing, pollen species are more abundant and exposure is greater in Baotou [20, 21]. A cross-sectional study by Li et al. [22] showed that in Chinese patients with asthma and/or rhinitis, house dust mites are the most common air allergen. In areas with long and cold winters, the incidence of house dust mite allergy is lower [23–25]. Inner Mongolia is located at a higher latitude than Beijing, and it has large day vs. night temperature difference. The average temperature in winter is approximately − 28 °C and the season can last for almost 6 months, which is not conducive to the growth and spread of house dust mites.

Among the AR population, the clinical symptoms of patients in Baotou area were more severe than those in Beijing. However, these differences were not observed in the NAR population. This may be due to the different etiologies of AR and NAR and the varying degrees of pollen exposure during the pollen season in the two regions. While mugwort pollen is the most important allergen for patients with AR in the pollen season for both regions, AR is an IgE-mediated type I allergic inflammation dominated by a type 2 immune response triggered by nasal mucosal contact with allergens. In contrast, NAR may be associated with neurogenic pathways, autonomic imbalance, age, pregnancy, occupation, or exposure to drugs [26], and does not vary based on seasonal changes in pollen counts. For individuals with NAR, symptoms are more likely to be perennial [27], explaining why in the pollen period studied, the clinical symptoms of the NAR population in Baotou were not classified as more severe than those in Beijing. Based on data regarding pollen concentration in 2019 that we obtained from two meteorological bureaus, it can be concluded that individuals in Baotou were exposed to pollen for a longer period of time. Furthermore, during the pollen period we selected for this study, the pollen concentration in Baotou was up to tenfold higher than that in Beijing, contributing to the significantly worse clinical symptoms experienced by patients with AR in Baotou when compared with those of patients in Beijing. Notably, although the major factor differentially affecting AR in grassland and non-grassland areas is allergen availability, epidemiological studies have shown that environmental triggers including risk factors (e.g., pollution) are considered major contributors to the dramatic increase in the incidence and the prevalence of allergic diseases, and the influence of these environmental factors is believed to be mediated by epigenetic mechanisms [28, 29]. Therefore, paying more attention to the impact of environmental factors will be the focus of our future work.

This study also analyzed the relevant factors affecting the severity of the overall clinical symptoms of patients with CR in the two regions; allergy and concomitant asthma were common risk factors for CR in both regions. AR and asthma often coexist as a combined airway disease [30]. Most patients with asthma experience symptoms of allergic inflammation in the airways. IgE plays a central role in the pathogenesis of various allergic diseases including asthma and AR [31, 32]; thus, patients with asthma tend to have more severe clinical symptoms. In addition, Artemisia pollen allergy is the main cause of asthma in northern China [6] and may explain why there are significantly more individuals with asthma in Baotou than in Beijing.

There were significant differences in the diagnosis, treatment, and symptom control of patients with CR in the two regions, due to the differing socio-economic status and medical level. On one hand, this may be attributed to the more severe clinical symptoms exhibited by patients with AR in Baotou. However, compared with those in Beijing, the government’s medical resources in Baotou are limited, which coexists with patients’ lack of awareness of the disease and poor patient compliance. In addition, allergen immunotherapy is currently the only therapeutic approach recommended by the World Health Organization that targets the cause of the disease and produces long-term effects through immune regulatory mechanisms [33]. Our findings showed that the number of patients receiving allergy immunotherapy in Baotou was significantly lower than that in Beijing, which was also associated with the prognosis of the disease. In Baotou, no corresponding allergy immunotherapy is currently being carried out for CR, and a small number of patients receive immunotherapy in hospitals in Beijing or other cities. Currently, immunotherapy is only available for use against dust mites in China. In Baotou, which is a representative grassland city, pollen is the most important allergen for the AR population. Therefore, more vigorous development of pollen allergen-specific immunotherapy is required in grassland cities for better long-term curative effects.

This study has its limitations. First, the Second Affiliated Hospital of Baotou Medical College and Beijing Tongren Hospital are both large-scale tertiary general hospitals, and most patients who visit these hospitals for treatment are urban residents. Access to care between urban and rural residents remains inconsistent. Second, we only included adult patients with CR in the study; pediatric patients were not included. Finally, in this study, we adopted the traditional standard for the sIgE antibody threshold, i.e., 0.35 kUA/L, to identify participants with a clinically evident allergic disease [15]. Although recent consensus guidance recommended that all clinical laboratories should report sIgE results as analytical measurements based on the accepted lower limit of quantitation of the assay [34], scholars have also emphasized that sIgE levels between 0.1 and 0.35 kUA/L should be cautiously interpreted, combined with the clinical symptoms after allergen exposure, because the clinical significance of the threshold remains ambiguous [35]. The proportion of patients with sIgE levels of 0.1–0.35 kUA/L in the pollen season collected in this study is extremely small, which may be related to the high exposure intensity of pollen in this period. Further research focusing on the correlation between sIgE levels lower than 0.35 kUA/L and the clinical symptoms in other periods, such as the winter season, is required. Future studies including a larger sample size and more detailed stratified sampling methods, especially including children, are required to further elucidate how pollen distribution and environmental factors contribute to the intensity of disease-related sensitization in order to develop more effective CR control strategies for different vegetation areas in North China.

## Conclusions

This study revealed differences in CR epidemiology between two regions in China by comparing and analyzing the status of patients with CR during the autumn pollen exposure period in Baotou and Beijing. There were significant differences in the allergen spectrum in the two cities. Allergy and combined asthma were common risk factors affecting the severity of clinical symptoms in the two regions. Compared with Beijing, patients with AR in Baotou present with more severe clinical symptoms that are not satisfactorily controlled due to the impact of pollen exposure and socio-economic and medical levels in the area.

## Supplementary Information

**Additional file 1. ** Questionnaire for patients with chronic rhinitis.

## Data Availability

All data generated or analyzed during this study are included in this published article.

## Abbreviations

CR: Chronic rhinitis
AR: Allergic rhinitis
NAR: Non-AR
ARIA: Allergic Rhinitis and its Impact on Asthma
sIgE: Specific-IgE
TNSS: Total nasal symptom score
PiAR: Pollen-induced AR
