# Supplementary material for: Comparative analysis of chronic rhinitis patient profiles during autumn pollen season between grassland and non-grassland cities in North China
Source: Allergy Asthma Clin Immunol. 2021 Oct 11;17:106. doi: 10.1186/s13223-021-00591-w (PMC8503993; doi:10.1186/s13223-021-00591-w)
Supplement: Supplementary file 1 — Additional file 1. Questionnaire for patients with chronic rhinitis. [file 13223_2021_591_MOESM1_ESM.docx]

**慢性鼻炎患者问卷调查表**

**姓名： 性别：男 / 女**

**年龄： 民族：**

**出生地： 现居住城市： （居住半年以上）**

**联系电话： 填写时间: 年 月 日**

**病史回顾：**（请您回答以下问题，并在相应选项前面的“□” 内划“√”）

1. **您的鼻部不适已持续了多长时间？**

年 月

1. **您的鼻部相关症状的发作时间：**

A、在一年内持续发作的时间：□ <4周 □ ≥4周

B、在一周内持续发作的时间：□ <4天 □ ≥4天

1. **在过去一年中，您的鼻部不适对您日常生活的总体困扰程度如何？**

A、睡眠正常：□ 是 □ 否

B、日常活动、体育锻炼、娱乐活动正常：□ 是 □ 否

C、工作和学习正常：□ 是 □ 否

D、有令人烦恼的症状：□ 是 □ 否

1. **是否伴有其他过敏性疾病？**

□ 无 □ 过敏性哮喘 □ 过敏性皮炎 □ 蚊虫叮咬过敏

□ 过敏性结膜炎 □ 药物过敏 □ 食物过敏（如海鲜、水果、牛奶等）

1. **您家族中是否有其他人有过敏性疾病？**

□ 无 □ 有，与您的关系是

1. **您是否伴有其它鼻腔疾病？**

□ 无 □ 慢性鼻窦炎 □ 鼻息肉 □ 鼻腔其他肿瘤 □ 其它

1. **您是否有吸烟史？**

□ 无 □吸烟，每日 支，持续 年

1. **您是否有饮酒史？**

□ 无 □饮酒，每日 两，持续 年

1. **您因为鼻部不适使用过以下哪些类药物(可多选):**
   - **口服抗组胺药**：息斯敏、氯雷他定（开瑞坦）、西替利嗪（仙特明/贝分）、盐酸非索非那定、依巴斯丁（开思亭）等。
   - **鼻用类固醇激素**：布地奈德（雷诺考特）、丙酸氟替卡松（辅舒良）、糠酸氟替卡松（内舒拿）等。
   - **口服激素**：甲泼尼龙（尤金/美卓乐）、醋酸泼尼龙等。
   - **抗生素**：头孢类、大环内酯类（阿奇霉素、克拉霉素、红霉素）、喹诺酮类（拜复乐）等。
   - **粘液促排剂**：切诺、欧龙马等。
   - **鼻腔冲洗**
2. **您是否接受过过敏免疫治疗？**

□ 否

□ 是，皮下脱敏治疗

□ 是，舌下脱敏治疗

1. **您是否因鼻部不适而接受手术治疗？**

□ 无

□ 有，共计手术 次

1. **您对目前已使用的治疗方式对的您的鼻部不适的整体控制情况如何评价？**

□ 4分：完全控制

□ 3分：实质控制

□ 2分：轻微控制

□ 1分：完全没有控制

□ 0分：完全没有控制甚至加重

1. **您过去一年因鼻部不适总共就诊几次：**

□ 0次

□ 1-5次

□ 6-10次

□ 10次以上

**症状评估：**

***♥*（如下图所示**，0~3表示您的相应症状的**严重程度**，请您根据**最近一年**的症状表现，在相应选项前面的“□” 内划“√”。**）**

**鼻堵：**

□ 0：无 □ 1：轻度 □ 2：中度 □ 3：重度

**鼻痒：**

□ 0：无 □ 1：轻度 □ 2：中度 □ 3：重度

**清水样涕：**

□ 0：无 □ 1：轻度 □ 2：中度 □ 3：重度

**打喷嚏：**

□ 0：无 □ 1：轻度 □ 2：中度 □ 3：重度

**谢谢您的认真填写！☺**

**Questionnaire for patients with chronic rhinitis**

**Name: Sex: Male/Female**

**Age: Ethnicity:**

**Birth place: City of residence: (Live for more than half a year)**

**Phone number: Fill in time: Day Month Year**

**Medical history review:**

(Please answer the following questions and mark “√” in the “□”in front of the corresponding option)

1. **How long has your nasal discomfort lasted?**

Month Year

1. **The onset time of your nose-related symptoms:**
2. Duration of onset within one year: □ < 4 weeks □ ≥4 weeks
3. Duration of onset within a week: □ < 4 days □ ≥4 days
4. **What is the general level of distress caused by your nose to your daily life in the past year?**
5. Normal sleep:

□ Yes □ No

1. Daily activities, physical exercises, and recreational activities are normal:

□ Yes □ No

1. Work and study are normal:

□ Yes □ No

1. Have troublesome symptoms:

□ Yes □ No

1. **Is it accompanied by other allergic diseases?**

□ No □ Asthma □ Allergic dermatitis

□ Mosquito bite allergy □ Allergic conjunctivitis □ Drug allergy

□ Food allergy (such as seafood, fruit, milk, etc.)

1. **Does anyone else in your family have allergic diseases?**

□ No □ Yes, their relationship with you is

1. **Do you have any other nasal diseases?**

□ No □ Chronic sinusitis □ Nasal polyps

□ Other tumors of the nasal cavity □ Other

1. **Do you have a history of smoking?**

□ No □Smoking, cigarettes/a day, last yeas

1. **Do you have a history of drinking?**

□ No □ Drinking, taels/a day, last yeas

1. **Which of the following medicines have you used due to nasal discomfort (multiple choices available)?**
   - **Oral antihistamines:** Aspismin, Loratadine (Carretan), Cetirizine (Zitermine/Befen), Fexofenadine hydrochloride, Ebastine (Carestine), etc.
   - **Nasal corticosteroids:** Budesonide (Ranocotte), Fluticasone propionate (Fushuliang), Fluticasone furoate (Nesunar), etc.
   - **Oral hormones:** Methylprednisolone (Eugene/Metrole), Acetate prednisolone, etc.
   - **Antibiotics:** Cephalosporins, Macrolides (Azithromycin, Clarithromycin, Erythromycin), Quinolones (Befole), etc.
   - **Mucus promoting agent:** Chernob, Ouloma, etc.
   - **Nasal irrigation**
2. **Have you received allergy immunotherapy?**

□ No

□ Yes, subcutaneous desensitization treatment

□ Yes, sublingual desensitization treatment

1. **Have you undergone surgery for nasal discomfort?**

□ No

□ Yes, surgeries in total

1. **How do you evaluate the overall control of your nasal discomfort with the current treatment methods?**

□ 4: Total control over symptoms

□ 3: Substantial control over symptoms

□ 2: Minor control over symptoms

□ 1: No control over symptoms

□ 0: Symptoms were aggravated

1. **How many times have you seen a doctor for nasal discomfort in the past year?**

□ 0 times

□ 1-5 times

□ 6-10 times

□ >10 times

**Symptom evaluation:**

***♥*** (In the figure below, 0–3 indicate the **severity** of your symptoms. Please mark “√” in the “□” in front of the corresponding option based on your symptoms **in the last year**.)

**Nasal congestion:**

□ 0: None □ 1: Mild □ 2: Moderate □ 3: Severe

**Nasal itching:**

□ 0: None □ 1: Mild □ 2: Moderate □ 3: Severe

**Runny nose:**

□ 0: None □ 1: Mild □ 2: Moderate □ 3: Severe

**Sneezing:**

□ 0: None □ 1: Mild □ 2: Moderate □ 3: Severe

**Thank you for filling this form in carefully! ☺**
